# Effectiveness of early essential newborn care implementation in four counties of western China

**DOI:** 10.1186/s12913-022-08570-6

**Published:** 2022-09-21

**Authors:** Chenran Wang, Yun Lin, Hanxiyue Zhang, Ge Yang, Kun Tang, Xiaobo Tian, Xiaona Huang, Tao Xu

**Affiliations:** 1 National Center for Women and Children’s Health, Chinese Center for Disease Control and Prevention, Haidian District, Room 601, No. 12 Da Huisi Rd, Beijing, 100081 People’s Republic of China; 2 Vanke School of Public Health, Tsinghua University, Beijing, China; 3 Division of Neonatology and Center for Newborn Care, Guangzhou Women and Children’s Medical Center, Guangzhou, China; 4 United Nations Children’s Fund Office for China, Beijing, China

**Keywords:** Early essential newborn care, Clinical practice, Western China

## Abstract

**Background:**

Neonatal survival is a public health concern globally. However, the regional disparity in neonatal mortality between rural counties of western China and urban areas of eastern provinces remains high. Early essential newborn care (EENC), recommended by World Health Organization, refers to a set of cost-effective interventions to improve neonatal health and development outcomes. In this study, we aimed to explore the effectiveness of EENC implementation in four counties of western China.

**Methods:**

Pre- and post-intervention investigations were conducted in four selected EENC intervention counties and four control counties of four western provinces of China, from June to August 2017 and from December 2020 to April 2021 respectively. A mixed quantitative and qualitative approach was used for data collection and analysis. Data on the coverage of EENC practices were collected via a post-intervention face-to-face questionnaire survey with postpartum mothers before hospital discharge. Hospital-reported data on neonatal health indicators were obtained through mail surveys in both investigations. We also performed semi-structured interviews with policymakers, health staff and postpartum mothers to understand their perceptions about the usefulness of EENC implementation.

**Results:**

Overall, 599 mother-newborn pairs in the intervention group and 699 pairs in the control group participated in the post-intervention survey. Controlling for the confounding factor of province, the proportion of newborns receiving EENC interventions was higher in the intervention group than in the control group (*P* < 0.05). Intervention groups in four provinces had higher coverage of: any skin-to-skin contact (99.50% *vs.* 49.07%); early breastfeeding initiation (within 60 min of birth) (90.84% *vs*. 80.35%); no medicine applied to the umbilical cord (98.50% *vs.* 9.73%); routine eye care (93.16% *vs.* 8.73%); and vitamin K_1_ administration (98.33% *vs.* 88.98%). EENC implementation was associated with decreased risk of neonatal diarrhea (*OR*: 0.326, 95% *CI*: 0.123, 0.865) and eye infection (*OR*: 0.147, 95% *CI*: 0.045, 0.483). Policymakers, health staff and postpartum mothers expressed satisfaction with the EENC interventions, noting a willingness among staff and policymakers to implement and sustain these interventions; the promotion of these interventions within hospital policy; the positive emotions experienced by postpartum mothers; perceived improvements in health; and improvements in support for health workers.

**Conclusion:**

EENC-recommended core practices (except kangaroo mother care) have been successfully introduced in pilot hospitals. The efficacy of EENC implementation should be highly recognized to accelerate the progress towards its national roll out.

**Supplementary Information:**

The online version contains supplementary material available at 10.1186/s12913-022-08570-6.

## Introduction

Neonatal health care is critical to child short- and long-term survival and development; ensuring a healthy start for all newborns will accelerate progress towards the Sustainable Development Goal (SDG) target of “ending preventable deaths of newborns by 2030” [1, 2]. While significant progress has been made in addressing child survival, neonatal death remains a serious concern globally, accounting for approximately 48% of all deaths among children under 5 years of age. More than two-thirds of neonatal deaths occur in the first three days after birth, especially in first 24 h after birth [3, 4]. China has reduced the rate of neonatal mortality from 33.1‰ in 1991 to 3.4‰ in 2020, however, newborn death is still the leading contributor to under-5 mortality [5]. Furthermore, the regional disparity in neonatal mortality between rural counties of western China and urban areas of eastern provinces remains high. The national report of China Maternal and Child Health Surveillance in 2020 showed that the neonatal mortality rates in western (4.8‰) and rural (3.9‰) regions were notably higher in comparison to eastern (1.8‰) and urban (2.1‰) areas [5]. The regional discrepancy in the burden of newborn deaths can be partly explained by the coverage inequality of high-quality newborn interventions across regions [6, 7]. Therefore, adopting effective interventions to address these discrepancies should be a high national priority.

Early Essential Newborn Care (EENC) is a package of evidence-based interventions for mothers and newborns delivered around birth. It was recommended by World Health Organization (WHO) *Action Plan for Healthy Newborn Infants in the Western Pacific Region (2014–2020)*to reduce preventable newborn deaths [8]. EENC emphasizes the minimization of unnecessary practices, such as routine suctioning and early physical examination for newborns, while promoting core cost-effective practices, including: immediate and uninterrupted mother-baby skin-to-skin contact (SSC) for at least 90 min, delayed umbilical cord clamping, early breastfeeding initiation, and kangaroo mother care for premature infants [9, 10]. All priority countries in the Western Pacific Region have continued to scale up EENC and the applicability of EENC has been substantiated in other countries [11]. To sustain these advancements, additional efforts are needed to integrate EENC into routine clinical practice.

With the aim of achieving equitable and high-quality coverage of health care for all newborns, the National Health Commission of China and the United Nations Children’s Fund (UNICEF) jointly launched the three-year Safe Neonatal Project in western China. From September 2017 to December 2020, the project was implemented in 18 counties of four western provinces with the highest burden of neonatal and under-5 deaths, including Qinghai, Sichuan, Guizhou, and Ningxia Hui Autonomous Region [7], setting out a vision of a nation where every newborn reaches his or her full potential. The purpose of the Safe Neonatal Project is to scale up EENC interventions in pilot counties; it aims to ensure that mothers and newborns in pilot areas increasingly benefit from equitable policies, guidelines, and quality, high-impact EENC practices for survival and development.

Understanding the health effects of EENC implementation is necessary to scale up EENC nationally. Evidence has indicated that EENC-recommended interventions are practical and cost-effective for improving neonatal health outcomes in western China [12, 13]. However, previous studies on EENC implementation were restricted to pre- and post-intervention design within groups [13, 14]; used only cross-sectional observational investigation of service capacity [15]; employed a narrow research scope with small sample size [16, 17]; and conducted limited qualitative research that lacked the perspectives of multiple stakeholders [18, 19]. Moreover, most studies lacked controls and a multi-centre design [13, 14, 16]. Our large sample, multi-centre study, is an attempt to address the above noted gaps in research to date. Based on data obtained from the Safe Neonatal Project, we aim to comprehensively explore the positive impacts of EENC implementation. Our findings will provide empirical references on the clinical practice of EENC to China and other countries with similar demands, with the goal of informing health authorities how tailored EENC promotion strategies can enhance neonatal health and well-being.

## Methods

### Study design

This was a pre- and post-intervention study. Data were collected from baseline and endline investigations, which were conducted from June to August 2017, and from December 2020 to April 2021, respectively. We conducted mixed quantitative and qualitative analyses to assess the positive impacts of EENC implementation.

### Study settings

Out of the 18 Safe Neonatal Project counties, one county was randomly selected as the intervention county in each province in our study, with a control county being selected from the same province. In each intervention/control county, one to two county-level hospitals providing midwifery services with over 1000 live births annually were selected as sample health facilities. A total of 15 health facilities were enrolled in this study, with seven in the intervention group and eight in the control group, respectively. The province-stratified group allocation and on-site survey time are shown in supplementary table 1. The two groups were homogeneous in socioeconomic development, demography, and maternal and child health care levels [20, 21].

### EENC implementation

#### EENC practices

EENC contains a package of evidence-based interventions for maternal and neonatal health improvement. The seven key practices are as follows:

1. Within 1 min after birth:
  a Neonatal resuscitation for newborns if without spontaneous breathing.
  b Immediate and prolonged SSC: The thoroughly dried neonate is in direct skin contact with mother’s bare breast and abdomen for ≥ 90 min. Cover (do not wrap) the newborn’s skin with clean warm cloth and the head with a hat.
2. Within 1–3 min of birth:
  iii Delayed umbilical cord clamping and proper care: Umbilical cord clamping is delayed until there is no umbilical pulsation. No sterilization medicines are applied to the cord stump.
3. Within 90 min of birth:
  iv Early breastfeeding initiation: Initiating early breastfeeding when baby presents feeding cues, including rooting, tonguing or biting hands. Baby latches on and stays fixed to the nipple, opening mouth widely to attach to mother’s breast and sucking successfully.
  v Kangaroo mother care for premature and low birthweight infants: Preterm newborns with gestational age ≤ 34 weeks or low birthweight newborns with birth weight ≤ 2000 g is placed in continuous SSC between his/her mother, father or other family members.
4. 90 min to 24 h after birth:
  f An intramuscular administration of 1 mg vitamin K_1_ to prevent neonatal hemorrhagic diseases.
  g Routine eye care with erythromycin to prevent neonatal eye infection.

Out of the core interventions, sustained SSC for ≥ 90 min, early breastfeeding initiation within 60 min after birth, no medicine applied to umbilical cord, routine eye care, and vitamin K_1_ administration were selected as key indicators to assess the general coverage of EENC practices in this study. Before EENC introduction in pilot counties, vitamin K_1_ administration had already been implemented in some of the enrolled health facilities.

#### Intervention group

EENC was introduced to the intervention counties through cascading coaching carried out through certified national and provincial facilitators. The coaching process strictly adhered to WHO *Early Essential Newborn Care Module 2—Coaching for the First Embrace—Facilitator’s Guide* [22]. Subsequently, the trained multidisciplinary team that was composed of obstetricians, midwives, pediatricians/neonatologists, nurses, and hospital administration (including infection control and quality assessment) staff implemented EENC-recommended practices following the publicly-published national expert consensus [23, 24].

The quality assessment of EENC implementation was carried out quarterly by national and provincial facilitators, in order to oversee the execution of EENC and ensure that all trained staff grasped the skills. Self-assessments of routine newborn care practices were recorded periodically by quality control teams of health facilities, and the assessment tools were formulated based on WHO *Early Essential Newborn Care Module 3—Introducing and sustaining EENC in hospitals: routine childbirth and newborn care* [23].

#### Control group

Non-recommended routine newborn health care practices, including immediate mother-newborn separation and umbilical cord clamping, cord wrapping, and application of disinfectant to the cord, were implemented in the control hospitals. Of note, EENC will be introduced to the control group when the study ends.

### Sample size estimation

#### Postpartum mother-newborn pairs

In the post-EENC phase, we conducted the questionnaire survey with postpartum mothers and their newborns before hospital discharge. Postpartum mothers and their newborns were selected based on the following criteria: 1) postpartum mothers agreed to participate this study with written informed consent; 2) mothers delivered vaginally at least two hours prior to questionnaire interviews; 3) mothers had not experienced a stillbirth or newborn death. Postpartum mothers who were multiparous were excluded to avoid duplication in data collection. The sample size of mother-newborn pairs was calculated using the following formula:

$$n=\frac{{\left[{Z}_{1-\alpha /2}\sqrt{2P\left(1-P\right)}+{Z}_{\beta }\sqrt{{P}_{1}\left(1-{P}_{1}\right)+{P}_{2}\left(1-{P}_{2}\right)}\right]}^{2}}{{\left({P}_{1}-{P}_{2}\right)}^{2}}$$

The proportion of early breastfeeding initiation was selected as the core indicator for estimating the sample size, where *P*_1_ is the proportion of early breastfeeding initiation (within 1 h after birth) before EENC implementation, and *P*_2_ is the proportion of early breastfeeding initiation after EENC implementation, and *P* is $\frac{{P_{1} + P_{2} }}{2}$_, and_*Z*_*1*_*-*_*α/2*_/*Z*_*β*_ is standard normal deviance at the significance level of *α*/1-*β.*

We assumed *P*_1_at baseline of 40% based on previous literature [25]. EENC implementation was expected to increase *P*_1_ by 20% and thus *P*_2_ was estimated as 60%. A minimum sample of 148 (*α* = 0.05; *β* = 0.10) mother–child pairs per group was therefore calculated with allowance for 15% invalid samples. The theoretical minimum sample size of mother-newborn pairs was 1184 (group*stratified province = 148*2*4).

Considering that the recruited hospitals from the geographically dispersed and health resource-limited western counties tended to have a low delivery volume annually, we enrolled all mother-newborn pairs fulfilling the enrollment criteria during the investigation, with the aim of ensuring an adequate sample size.

#### Interested stakeholders

During the endline investigation, we conducted semi-structured focus group discussions with policymakers and health staff, and individual in-depth interviews with postpartum mothers in each group of the eight counties, as they represented different roles of stakeholders regarding the EENC intervention. National policies, WHO EENC modules, and relevant literature were reviewed to help synthesize interviews. We performed purposive sampling to recruit participants and the sample size was determined as per data saturation.

### Data collection

#### Mail survey for health facilities

Mail surveys were conducted in all 15 enrolled health facilities to review the impact of EENC on newborn health indicators before and after implementation. The mail survey questionnaire was designed based on WHO *Early Essential Newborn Care Module 1- Annual implementation review and planning guide.* It collected neonatal health indicators in the past 12 months, which were recorded by directors of obstetrics or neonatology/pediatrics based on routine monitoring records of the recruited health facilities. The same electronic questionnaires were issued by the National Health Commission of China at both baseline and endline phases, while data submission was overseen and verified by hospital quality control teams. Health indicators of interest included neonatal diarrhea, umbilical infection, eye infection, and mortality during the neonatal period (0 ~ 28 days).

#### Questionnaire survey for mother-newborn pairs

The questionnaire for postpartum mothers and newborns was designed in reference to the WHO *Early Essential Newborn Care Module 3* [19] and focused on coverage indicators of EENC key practices. Specifically, data on SSC and breastfeeding practices were obtained from face-to-face questionnaire interviews with postpartum mothers, while coverage on practices of umbilical cord and routine eye care, and vitamin K_1_ administration were extracted from neonatal medical records. The contents of questionnaire survey were documented by midwives, obstetric nurses and doctors.

#### Qualitative survey with interested stakeholders

The qualitative survey focused on the knowledge of EENC, adherence to the EENC-recommended practices, and target populations’ satisfaction of EENC implementation. All interviews were conducted in quiet and private rooms to enable stakeholders to provide subjective assessments of EENC implementation according to their experiences. Every focus group discussion and in-depth interview lasted for approximately 90 min and 30 min, respectively. All participants involved in interviews provided written informed consent.

### Neonatal health indicators of interest

The effectiveness of EENC implementation was evaluated by the quantification of differences in core practices coverage and neonatal health indicators (neonatal diarrhea, umbilical infection, eye infection, and mortality during the neonatal period) between intervention and control groups, and perceived usefulness of the interventions obtained from interested stakeholders.

### Data analysis

Data obtained from mail surveys and questionnaire surveys were in institutional and individual level forms, respectively. As our study was conducted in four provinces, stratification by province was performed to control the potential regional variation. We did descriptive analyses of demographic characteristics of postpartum mothers and babies with frequency (proportion) and mean ± standard deviance (SD). Cochran-Mantel–Haenszel (CMH) test/Fisher exact probability method for categorical variables and *t*-test for quantitative data, with the confounding factor being controlled for, were carried out to test the significance of differences between intervention and control groups. We used 95% confidence intervals (*CI*) to estimate the uncertainty in differences of EENC practices coverage between the two groups. Unconditional logistic model was used to examine the association between EENC implementation and neonatal health indicators. Participants with missing data for key variables were removed. Data were put into EpiData 3.1 with double-entry method, and SAS 9.4 (SAS Institute Inc., Cary, NC, USA) was used to conduct statistical analyses. The significance level of *α* was 0.05.

All interviews were audio-taped and the recordings were transcribed into textual materials. The thematic framework approach was used to analyse qualitative data. Perspectives of multiple stakeholders (i.e., policymakers, health staff and postpartum mothers) on each thematic category were generalized and presented for mutual authentication. The four stages of data analysis were: [26] (1) familiarization with transcripts; (2) identification of a thematic framework; (3) data coding; and (4) interpretation of main findings. QSR Nvivo 12.0 was used for data generalization and numerical coding.

## Results

### Demographic characteristics of mother-newborn pairs

A total of 1373 questionnaires of mother-newborn pairs were collected in the endline survey, of which 75 were invalid due to inappropriate subjects, and excessive missing items or logic errors. Finally, we enrolled 1298 mother-newborn pairs from eight counties in the four provinces, with 599 pairs in the intervention group and 699 pairs in the control group. With the confounding factor of province being controlled for, no statistically significant differences were found in the distribution of maternal age and neonatal birthweight (*P* > 0.05). Pregnant women in the intervention group had an average age of 26.11 ± 5.44 years, and 383 (63.94%) had an educational attainment of junior high school or lower; postpartum mothers in the control group were aged 26.33 ± 5.18 years and 423 (60.52%) had junior high school level education or lower. Newborns enrolled in the intervention and control groups had mean birth lengths of 49.59 ± 1.76 cm and 50.33 ± 1.71 cm, with 570 (95.16%) and 653 (93.42%) weighing 2500 ~ 4000 g, respectively. Demographic characteristics of recruited mother-newborn pairs were shown in Table 1.

**Table 1:** Demographic characteristics of postpartum mothers and newborns according to endline survey (*N* = 1298)

	Total (*N* = 1298)				Guizhou Province (n = 421)				Qinghai Province (*n* = 280)	
	Intervention (*n* = 599)	Control (*n* = 699)	*t*/*χ*^2^	*P*	Intervention (*n* = 150)	Control (*n* = 271)	*t*/*χ*^2^	*P*	Intervention (*n* = 137)	Control (*n* = 143)
**Maternal**										
Age ($\overline{x }$±SD)	26.11 ± 5.44	26.33 ± 5.18	0.766^#^	0.444	24.16 ± 6.10	25.73 ± 5.84	2.596	0.010	26.37 ± 5.12	26.14 ± 5.20
Education level			14.096^#^	0.003			13.028	0.005		
Primary school or below	169 (28.21)	149 (21.32)			37 (24.67)	33 (12.18)			77 (56.20)	85 (59.44)
Junior high school	214 (35.73)	274 (39.20)			85 (56.67)	164 (60.52)			22 (16.06)	21 (14.69)
Senior high school	110 (18.36)	154 (22.03)			18 (12.00)	40 (14.76)			10 (7.30)	20 (13.99)
College or higher	106 (17.70)	122 (17.45)			10 (6.67)	34 (12.55)			28 (20.44)	17 (11.89)
Nationality			22.276^#^	< 0.001			42.334	< 0.001		
Minorities	298 (49.75)	284 (40.63)			90 (60.00)	75 (27.68)			136 (99.27)	128 (89.51)
Han nationality	301 (50.25)	415 (59.37)			60 (40.00)	196 (72.32)			1 (0.73)	15 (10.49)
Parity			7.490^#^	0.024			0.537	0.765		
First birth	216 (36.06)	228 (32.62)			39 (26.00)	73 (26.94)			61 (44.53)	48 (33.57)
Second birth	194 (32.39)	265 (37.91)			42 (28.00)	83 (30.63)			42 (30.66)	60 (41.96)
Third birth or above	189 (31.55)	206 (29.47)			69 (46.00)	115 (42.44)			34 (24.82)	35 (24.48)
**Infants**										
Birthweight (g)			2.673^#^	0.263			—	0.350^*^		
< 2500	13 (2.17)	26 (3.72)			3 (2.00)	10 (3.69)			5 (3.65)	6 (4.20)
2500 ~ 4000	570 (95.16)	653 (93.42)			145 (96.67)	252 (92.99)			127 (92.70)	133 (93.01)
> 4000	16 (2.67)	20 (2.86)			2 (1.33)	9 (3.32)			5 (3.65)	4 (2.80)
Birth length (cm) ($\overline{x }$±SD)	49.59 ± 1.76	50.33 ± 1.71	7.699	< 0.001	50.41 ± 1.90	50.01 ± 0.69	3.086	0.002^**^	49.62 ± 2.10	51.00 ± 2.00
Gestational age			6.443^#^	0.011			5.689	0.017		
Full-term birth	589 (98.33)	670 (95.85)			149 (99.33)	257 (94.83)			133 (97.08)	140 (97.90)
Premature/ Low birthweight	10 (1.67)	29 (4.15)			1 (0.67)	14 (5.17)			4 (2.92)	3 (2.10)

	Qinghai Province (*n* = 280)			Sichuan Province (*n* = 282)			Ningxia Hui Autonomous Region (*n* = 315)			
	*t*/*χ*^2^	*P*	Intervention (*n* = 152)	Control (*n* = 130)	*t*/*χ*^2^	*P*	Intervention (*n* = 160)	Control (*n* = 155)	*t*/*χ*^2^	*P*
**Maternal**										
Age ($\overline{x }$±SD)	-0.377	0.707	26.57 ± 4.40	26.57 ± 4.04	0.202	0.840	27.37 ± 5.51	27.38 ± 4.63	0.021	0.984^**^
Education level	6.315	0.097			—	0.002^*^			7.939	0.047
Primary school or below			5(3.29)	2 (1.54)			50 (31.25)	29 (18.71)		
Junior high school			58 (38.16)	25 (19.23)			49 (30.63)	64 (41.29)		
Senior high school			59(38.82)	67(51.54)			23 (14.38)	27 (17.42)		
College or higher			30 (19.74)	36 (27.69)			38 (23.75)	35 (22.58)		
Nationality	12.370	< 0.001			—	0.461^*^			1.379	0.240
Minorities			0 (0.00)	1 (0.77)			72 (45.00)	80 (51.61)		
Han nationality			152(100.00)	129 (99.23)			88 (55.00)	75 (48.39)		
Parity	4.615	0.100			19.408	< 0.001			0.861	0.650
First birth			61 (40.13)	61 (46.92)			55 (34.38)	46 (29.68)		
Second birth			54 (35.53)	62 (47.69)			56 (35.00)	60 (38.71)		
Third birth or above			37 (24.34)	7 (5.38)			49 (30.63)	49 (31.61)		
**Infants**										
Birthweight (g)	—	0.940^*^			—	0.351^*^			—	0.402^*^
< 2500			3 (1.97)	5 (3.85)			2 (1.25)	5 (3.23)		
2500 ~ 4000			143 (94.08)	123 (94.62)			155 (96.88)	145 (93.55)		
> 4000			6 (3.95)	2 (1.54)			3 (1.88)	5 (3.23)		
Birth length (cm) ($\overline{x }$±SD)	5.635	< 0.001^*^	50.14 ± 1.48	49.85 ± 0.91	-1.907	0.058	48.65 ± 1.98	49.97 ± 1.32	6.986	< 0.001^**^
Gestational age	0.003^§^	0.954			2.553^§^	0.110			0.586^§^	0.444
Full-term birth			150 (98.68)	123(94.62)			157 (98.13)	150 (96.77)		

^*^ Fisher exact probability

^**^ Satterthwaite t' test (heterogeneous variance)

^#^Cochran-Mantel–Haenszel (CMH) with controlled variable as province

### Coverage of EENC core interventions

**Fig. 1:**
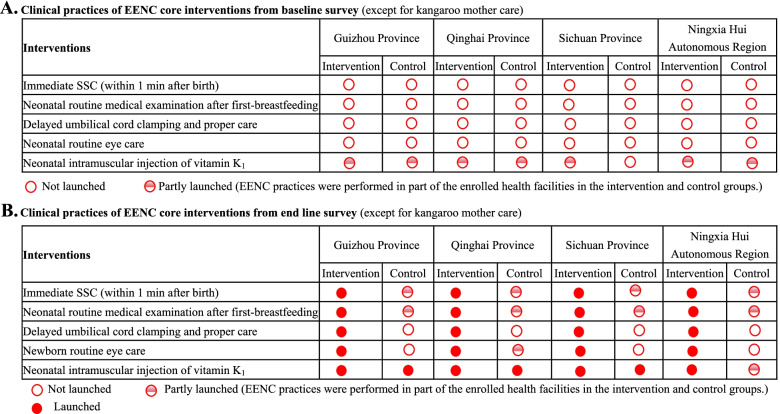
Clinical practices of EENC core interventions (except for kangaroo mother care)

Before the introduction of EENC, other key clinical practices were not performed in both intervention and control groups (with the exception of vitamin K_1_ administration, which had been implemented in some of the enrolled health facilities). All practices, except for kangaroo mother care, were implemented in intervention counties after EENC implementation. SSC and routine eye care were partly implemented in control groups (Fig. 1).

With the confounding variable of province being controlled for, compared with the control group, significant improvements in proportions of neonates receiving all selected EENC core practices were noted in the intervention group at end line (*P* < 0.05). The disparities in practice coverage between the two groups were homogeneous in four provinces. Intervention groups in four provinces had higher coverage of immediate SSC (within 1 min) (85.07% *vs*. 21.87%); prolonged SSC for at least 90 min (78.02% *vs*. 0.58%); early breastfeeding initiation (within 60 min) (90.84% *vs*. 80.35%); and exclusive breastfeeding before discharge (92.57% *vs*. 63.80%). Coverage was particularly high in Guizhou Province: 92.62% for immediate SSC; 90.60% for prolonged SSC; 97.98% for early breastfeeding initiation; and 95.30% for exclusive breastfeeding. In the intervention groups, no applied medicine to the umbilical cord (98.50% *vs*. 9.73%), routine eye care (93.16% *vs*. 8.73%), and intramuscular injection of vitamin K_1_ (98.33% *vs*. 88.98%) were common (> 90%), except for routine eye care in Ningxia Hui Autonomous Region (Table 2).

**Table 2:** Coverage of EENC core interventions in four provinces according to endline survey (*N* = 1298)

	Total (*N* = 1298)				Guizhou Province (*n* = 421)				Qinghai Province (*n* = 280)	
	Intervention (*n* = 599)	Control (*n* = 699)	*t*/*χ*^2^	*P*	Intervention (*n* = 150)	Control (*n* = 271)	*t*/*χ*^2^	*P*	Intervention (*n* = 137)	Control (*n* = 143)
**SSC**			463.344^#^	< 0.001			368.234	< 0.001		
Any SSC	596 (99.50)	343 (49.07)			149 (99.33)	12 (4.43)			137 (100.00)	61 (42.66)
Time of SSC initiation post birth (min)			331.742^#^	< 0.001			—	< 0.001^*^		
< 1	507 (85.07)	75 (21.87)			138 (92.62)	0 (0.00)			88 (64.23)	60 (98.36)
1 ~ 10	89 (14.93)	211 (61.52)			11 (7.38)	5 (41.67)			49 (35.77)	0 (0.00)
11 ~ 59	0 (0.00)	36 (10.50)			0 (0.00)	5 (41.67)			0 (0.00)	1 (1.64)
≥ 60	0 (0.00)	21 (6.12)			0 (0.00)	2 (16.67)			0 (0.00)	0 (0.00)
SSC duration (min)			535.143^#^	< .0001			—	< 0.001^*^		
< 10	15 (2.52)	136 (39.65)			2 (1.34)	7 (58.33)			3 (2.19)	24 (39.34)
10 ~	51 (8.56)	132 (38.48)			0 (0.00)	2 (16.67)			23 (16.79)	29 (47.54)
30 ~	40 (6.71)	70 (20.41)			9 (6.04)	3 (25.00)			2 (1.46)	7 (11.48)
60 ~	25 (4.19)	3 (0.87)			3 (2.01)	0 (0.00)			1 (0.73)	0 (0.00)
≥ 90	465 (78.02)	2 (0.58)			135 (90.60)	0 (0.00)			108 (78.83)	1 (1.64)
**Breastfeeding**										
Successful first-time breastfeeding with provision of SSC	523 (87.75)	270 (78.72)	2.381^#^	0.123	147 (98.66)	5 (41.67)	—	< 0.001^*^	133 (97.08)	61 (100.00)
Any breastfeeding	579 (96.66)	641 (91.70)	12.823^#^	< 0.001	149 (99.33)	238 (87.82)	17.232	< 0.001	135 (98.54)	140 (97.90)
Initiation (min)			25.898^#^	< 0.001			34.615	< 0.001		
≤ 60	526 (90.84)	515 (80.35)			146 (97.99)	180 (75.63)			134 (99.26)	105 (75.00)
61 ~ 90	32 (5.53)	67 (10.45)			0 (0.00)	10 (4.20)			1 (0.74)	30 (21.43)
> 90	21 (3.63)	59 (9.20)			3 (2.01)	48 (20.17)			0 (0.00)	5 (3.57)
Duration (min)			47.046^#^	< 0.001			47.243	< 0.001		
< 10	80 (13.82)	62 (9.67)			6 (4.03)	27 (11.34)			62 (45.93)	17 (12.14)
10 ~	178 (30.74)	221 (34.48)			47 (31.54)	79 (33.19)			71 (52.59)	32 (22.86)
20 ~	73 (12.61)	172 (26.83)			19 (12.75)	81 (34.03)			0 (0.00)	55 (39.29)
30 ~	248 (42.83)	186 (29.02)			77 (51.68)	51 (21.43)			2 (1.48)	36 (25.71)
Exclusive breastfeeding	536(92.57)	409(63.80)	131.476^#^	< 0.001	142 (95.30)	96 (40.34)	116.922	< 0.001	133 (98.52)	112 (80.00)
**No applied medicine to the umbilical cord**	590 (98.50)	68 (9.73)	1042.438^#^	< 0.001	147 (98.00)	1 (0.37)	403.733	< 0.001	134 (97.81)	65 (45.45)
**Routine eye care**	558(93.16)	61(8.73)	965.278^#^	< 0.001	150 (100.00)	0 (0.00)	421.000	< 0.001	135(98.54)	59(41.26)
**Intramuscular injection of vitamin K**_**1**_	589(98.33)	622(88.98)	71.015^#^	< 0.001	149(99.33)	270 (99.63)	0.000^§^	1.000	137 (100.00)	142 (99.30)

	Qinghai Province (*n* = 280)		Sichuan Province (*n* = 282)				Ningxia Hui Autonomous Region (*n* = 315)			
	*t*/*χ*^2^	*P*	Intervention (*n* = 152)	Control (*n* = 130)	*t*/*χ*^2^	*P*	Intervention (*n* = 160)	Control (*n* = 155)	*t*/*χ*^2^	*P*
**SSC**	111.094	< 0.001			8.959^§^	0.003			4.208^§^	0.040
Any SSC			151 (99.34)	121 (93.08)			159 (99.38)	149 (96.13)		
Time of SSC initiation post birth (min)	—	—			—	< 0.001^*^			244.389^§^	< 0.001
< 1			132 (87.42)	8 (6.61)			149 (93.71)	7 (4.70)		
1 ~ 10			19 (12.58)	91 (75.21)			10 (6.29)	115 (77.18)		
11 ~ 59			0 (0.00)	17 (14.05)			0 (0.00)	13 (8.72)		
≥ 60			0 (0.00)	5 (4.13)			0 (0.00)	14 (9.40)		
SSC duration (min)	113.372	< 0.001			233.929	< 0.001			167.786	< 0.001
< 10			2 (1.32)	44 (36.36)			8 (5.03)	61 (40.94)		
10 ~			2 (1.32)	25 (20.66)			26 (16.35)	76 (51.01)		
30 ~			4 (2.65)	50 (41.32)			25 (15.72)	10 (6.71)		
60 ~			16 (10.60)	1 (0.83)			5 (3.14)	2 (1.34)		
≥ 90			127 (84.11)	1 (0.83)			95 (59.75)	0 (0.00)		
**s**										
Successful first-time breastfeeding with provision of SSC	0.642^§^	0.423	148 (98.01)	103 (85.12)	15.664	< 0.001	95 (59.75)	101 (67.79)	2.147	0.143
Any breastfeeding	0.000^§^	1.000	143 (94.08)	119 (91.54)	0.686	0.407	152 (95.00)	144 (92.90)	0.611	0.435
Initiation (min)	35.569	< 0.001^*^			2.691	0.260			13.737	< 0.001
≤ 60			134 (93.71)	106 (89.08)			112 (73.68)	124 (86.11)		
61 ~ 90			3 (2.10)	7 (5.88)			28 (18.42)	20 (13.89)		
> 90			6 (4.20)	6 (5.04)			12 (7.89)	0 (0.00)		
Duration (min)	125.772	< 0.001			—	< 0.001			6.432	0.092
< 10			0 (0.00)	9 (7.56)			12 (7.89)	9 (6.25)		
10 ~			10 (6.99)	42 (35.29)			50 (32.89)	68 (47.22)		
20 ~			16 (11.19)	6 (5.04)			38 (25.00)	30 (20.83)		
30 ~			117 (81.82)	62 (52.10)			52 (34.21)	37 (25.69)		
Exclusive breastfeeding	24.250	< 0.001	127 (88.81)	96 (80.67)	3.396	0.065	134 (88.16)	105 (72.92)	11.048	0.001
**No applied medicine to the umbilical cord**	93.283	< 0.001	150 (98.68)	2 (1.54)	266.132	< 0.001	159 (99.38)	0 (0.00)	311.025	< 0.001
**Routine eye care**	107.880	< 0.001	152 (100.00)	0 (0.00)	282.000	< 0.001	121 (75.63)	2 (1.29)	182.784	< 0.001
**Intramuscular injection of vitamin K**_**1**_	0.000^§^	1.000	152 (100.00)	130 (100.00)	—	—	151 (94.38)	80 (51.61)	73.619	< 0.001

^*^: Fisher exact probability

^#^: Cochran-Mantel–Haenszel (CMH) with controlled variable of province

^§^: Corrected chi-squared test

**Fig. 2:**
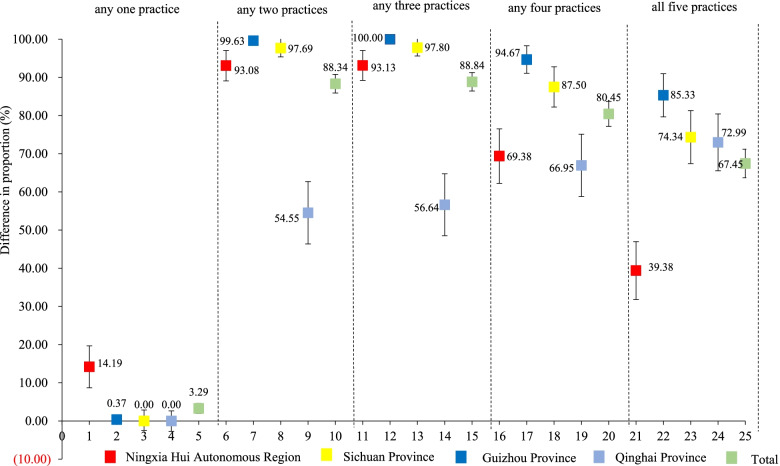
Difference in general coverage of EENC core interventions between intervention and control groups according to endline survey

More babies in the intervention group versus control group received five EENC practices post intervention, with the difference in coverage between the two groups being 67.45% (95% *CI*: 63.69%–71.20%). Differences in the proportion of newborns receiving all five practices between two groups in Guizhou, Qinghai, Sichuan and Ningxia were 85.33% (79.67%–90.99%), 72.99% (65.56%–80.43%), 74.34% (67.40% ~ 81.29%), and 39.38% (31.80%–46.95%), respectively (Fig. 2).

The differences in coverage of EENC practices between the two groups in detail were shown in supplementary Figs. 1, 2, 3, 4, 5, 6, 7, 8, 9,10,11, and supplementary Table 2.

### Neonatal health indicators

The univariate logistic regression model showed that EENC implementation was associated with decreased risk of neonatal diarrhea (*OR*: 0.327, 95% *CI*: 0.123, 0.867) and eye infection (*OR*: 0.147, 95% *CI*: 0.045, 0.483). After controlling for the confounding factor of province, similar results were found in association between EENC and decreased risk of neonatal diarrhea (*OR*: 0.326, 95% *CI*: 0.123, 0.865) and eye infection (*OR*: 0.147, 95% *CI*: 0.045, 0.483). The result was not powered enough to detect the potential association between EENC implementation and neonatal mortality and umbilical infection (Table 3).

**Table 3:** The association between EENC implementation and neonatal health indicators

	Model 1				Model 2^a^			
	*OR*	95% *CI*	$S_{{\overline{X} }}$	*P*	*OR*	95% *CI*	$S_{{\overline{X} }}$	*P*
Neonatal diarrhea	0.327	0.123, 0.867	0.498	0.025	0.326	0.123, 0.865	0.498	0.024
Umbilical infection	2.292	0.547, 9.592	0.731	0.256	2.278	0.544, 9.539	0.731	0.260
Eye infection	0.147	0.045, 0.483	0.608	0.002	0.147	0.045, 0.483	0.608	0.002
Neonatal mortality	0.393	0.082, 1.890	0.802	0.244	0.391	0.081, 1.884	0.802	0.242

^a^ controlled variable: province

### EENC satisfaction of stakeholders

183 participants recruited from eight counties were interviewed, consisting of 52 policymakers (25 in the intervention group and 27 in the control group); 94 health staff members (45 in the intervention group and 49 in the control group); and 37 postpartum mothers (15 in the intervention group and 22 in the control group). The sample size of interviewees recruited in each group stratified by province is reported in supplementary Table 3.

The satisfaction level of interested parties was summarized into six sub-themes: recognition, policy, emotion, work support, health outcomes, and sustainability (see supplementary Fig. 12). Most stakeholders stated that EENC had been widely recognized. The positive health effects of EENC practices promoted the implementation of policies relevant to newborn health care at the county and hospital levels. SSC brought great happiness to mothers and helped them bond with their newborns. Health staff stated that the EENC coaching improved their professional skills and promoted the normalization and standardization of clinical practices. Notably, some participants believed that EENC implementation was associated with reduced incidence risk of neonatal hypothermia, umbilical cord infection and neonatal mortality. Because EENC brought direct health benefits to health workers, mothers, and neonates, most policymakers clearly expressed their commitment to continue to implement EENC-recommended interventions. Selected quotes from stakeholders regarding satisfaction with EENC interventions are reported in Table 4.

**Table 4:** Examples of stakeholder attitudes regarding satisfaction of EENC implementation

Theme category	Main findings	Quotations
**Satisfaction**		
Recognition	EENC-recommended practices gained wide acceptance of policymakers and health staff	"Positive feedback from the newborn’s family members means that EENC is acceptable and applicable." (a policymaker in the intervention group of Sichuan Province)
		"We are highly willing to implement EENC practices. After the EENC implementation, we are delighted to observe the health benefits, and our traditional conceptions have changed. Particularly, SSC, umbilical cord care, as well as eye care, bring benefits not merely to us, but also to mothers and newborns." (a health staff in the intervention group of Sichuan Province)
Policy	The positive effects of EENC implementation promoted the establishment of principles on neonatal health at both county and hospital levels	"We issued some health policies to promote EENC. For example, the 'Safe Neonatal Project' has been listed to our 13th Five-Year Plan. In the next five years, we will continuously incorporate this project into the 14th Five-Year Plan, as the focus of newborn health improvement." (a policymaker in the intervention group of Guizhou Province)
Emotion	EENC practices, particularly SSC, improved mothers' happiness	"One mother said: it is really such a great feeling! I am so happy!" (a health staff in the intervention group of Sichuan Province)
	SSC strengthened parent–child bonding	"My baby was put on my breast immediately after birth, and there was a good sense of intimate attachment between by little baby and me. I felt so excited and happy. (Interviewer: Is this feeling different from your previous two babies? Mother: I feel more intimate with this one)." (a postpartum mother receiving SSC in the control group of Qinghai Province)
Health outcomes	EENC implementation was perceived to be associated with reduced incidence risk of neonatal hypothermia and umbilical cord infection, and neonatal mortality	"Before EENC introduction, newborns were separated from their mothers after delivery, and the incidence risk of hypothermia was quite high. However, hypothermia incidents have decreased considerably since EENC practices were implemented among pregnant women who delivered vaginally in 2018." (a health staff in the intervention group of Ningxia Hui Autonomous Region)
		"I notice that after EENC implementation, the incidence risk of umbilical infection has been reduced distinctly, and the occurrence is scarcely observed." (a health staff in the intervention group of Qinghai Province)
Sustainability	Most interviewees expressed their determination to continue to implement EENC-recommended practices	"EENC-recommended practices should be further scaled up in our poverty-stricken areas." (a health staff in the intervention group of Ningxia Hui Autonomous Region)
Work support	The implementation of EENC provides support for health workers, including improvement of professional skills, standardization of vital clinical practices, and conservation of medical consumables	"Some practices, including eye care, and vitamin K_1_ administration, are simple and easy to operate, which may partly explain the reason for the successful scale-up in our pilot hospital."(a health staff in the intervention group of Ningxia Hui Autonomous Region)
		"EENC promotes exclusive breastfeeding, which reduces neonatal diarrhea and allergies induced by breastfeeding substitutes. The health effects in turn alleviate our routine workload." (a health staff in the intervention group of Guizhou Province)

## Discussion

To the best of our knowledge, this is the first and largest study to investigate the coverage and health effects of EENC practices in western China. According to our present study, a considerably higher coverage of EENC key practices, including immediate and prolonged SSC, early breastfeeding initiation, no medicine applied to the umbilical cord, eye care, and vitamin K_1_administration, was observed in intervention hospitals in the post-EENC phase. Furthermore, we found that compared with our interim evaluation in the same provinces [13], the proportions of newborns receiving EENC key practices increased (SSC duration ≥ 90 min: 63.1% *vs*.78.0%; any breastfeeding: 96.1% *vs*. 96.7%; vitamin K_1_ administration: 79.7% *vs*. 98.3%; newborn eye care: 58.4% *vs*. 93.2%), which indicates the successful EENC promotion in pilot counties. However, kangaroo mother care for preterm newborns was not implemented in intervention hospitals due to limited technical support in western counties. These findings provide important information for optimizing EENC practices further in poverty-stricken areas of western China, and potentially in other high-demand areas of the world.

Our study showed that mother-newborn SSC was associated with increased proportions of early initiation breastfeeding and a longer duration of first-breastfeeding – findings that are highly consistent with previous studies [27, 28]. SSC may activate the oxytocinergic system, and the elevated concentration of oxytocin in postpartum mothers in turn promotes lactation and prolonged breastfeeding duration [29]. In response to the WHO recommendation to “*facilitate immediate and uninterrupted skin-to-skin contact and support mothers to initiate breastfeeding as soon as possible after birth*”, advocated as part of the Baby-Friendly Hospital Initiative [30], SSC should be steadily facilitated to provide optimal breastfeeding support and high-quality services for mothers and newborns. Additionally, we found that SSC was a positive experience for most postpartum mothers that improved mothers’ satisfaction, breastfeeding confidence, and mother–child bonding. As WHO indicates, high-quality health care can encourage mothers to adopt pro-health behaviours [31]. Further, in Viet Nam, some private hospitals regarded EENC as a special service that attracted pregnant women seeking high-quality antenatal and childbirth care [18]. To optimize the experience of postpartum mothers and improve the quality of health services, efforts should be taken to make SSC available to all mothers and neonates.

The usual practices of clamping the umbilical cord immediately after birth and applying disinfectant to the cord can increase the risk of bacterial infection [22]. The guideline released by WHO in 2014 indicated that, for improved maternal and child health outcomes, umbilical cord clamping should be delayed until there is no cord pulsation [32]. Indeed, a quasi-experimental study in China found that newborns receiving EENC interventions experienced lower umbilical cord infection rate compared to those in control groups (0.3% *vs*. 0.9%) [33]; however, no significant health effect was found in our study. Even though the proportion of newborns receiving no applied medicine to the umbilical cord was high in pilot hospitals, the lower umbilical infection rate (0.43% *vs*. 0.00%) was observed merely in pilot hospitals of Sichuan Province. The undetected health effects in other centres may be partly explained by the imperfect quality of hospital-reported data, which highlights the need for more stringent quality control measures and routine health management in pilot health facilities.

WHO-recommended routine eye care and vitamin K_1_administration should be applied to prevent neonatal ophthalmia and haemorrhage [34, 35], and the health benefits of this practice have been reported in previous publications [35–37]. Although the overall eye infection rate in the intervention group was found lower than the control counterpart (0.04% *vs*. 0.29%), further studies with longer observation period and more rigorous quality supervision are needed to strengthen the evidence base.

Normalization Process Theory provides a theoretical framework for assessing the probability of routine embedding of complex interventions: when practitioners acknowledge the importance and benefits of new interventions, the new routine is expected to be sustainable [38]. Consistent with Normalization Process Theory, we observed that the positive feedback from postpartum mothers and improved neonatal health indicators convinced health staff of the value of EENC, which in turn motivated them to routinely implement EENC- recommended practices. Our findings contribute to the growing evidence for introducing these effective practices into primary health facilities.

Nevertheless, previous studies have noted the lack of national technical guidelines for newborn care in China, which have caused inconsistencies in childbirth and early newborn care practices across regions [39]. As such, it is anticipated to be an uphill battle to scale up EENC nationwide in China. Moreover, Frederick AC et al. revealed that health staff shortages were also perceived as a challenge to the implementation of EENC practices in our pilot hospitals, leading to interruptions in health worker support for sustained SSC. [18, 40] Since regional disparities between western rural counties and urban areas in economically developed provinces in China remain high, the allocations in equipment supplied to western health facilities should be increased (e.g., maternity wards, radiant warmers, and neonatal resuscitation equipment) [41, 42]. In our present study, most postpartum mothers had junior high school or lower education. Current evidence suggests that education level can be one of the most important socioeconomic factors in gaining access to EENC practices. Better access to education may be associated with the increased availability of high-quality health services, while the health behaviours of poorly educated mothers were easily affected by their surroundings [43, 44]. Therefore, increased investment in timely and effective promotion of EENC, such as the provision of accessible health educational materials, should be positioned as a priority for poorly educated pregnant women and their family members in poverty-stricken or geographically distant areas.

This is the first study to evaluate the effectiveness of EENC implementation through various data sources and mixed quantitative and qualitative methods. By introducing the control group, our findings indicate that EENC implementation is associated with improved health status of neonates, and the differences in core practice coverage between groups needed to be considered in the further scale-up of EENC. Our results can be regarded as a valuable reference on further promotion of EENC in China and other countries with a similar context. Compared with previous studies with pre- and post-intervention design, the control group was set in our study to better assess the improvement of EENC core intervention coverage and health indicators. Considering the potential regional variation across four western provinces, we conducted stratification analyses, controlling for the confounding variable of province. Moreover, the extraction of some EENC key coverage indicators from neonatal medical records helps to alleviate observation bias (i.e., the Hawthorne effect). Notably, we collected assessments of multiple stakeholders, including policymakers, health staff, and postpartum mothers on each thematic category, which provided mutual authentication across different roles. In our study, the quantitative and qualitative results validated and complemented each other, which enhanced the validity of our findings.

This study has some limitations. Firstly, we collected questionnaire data on postpartum mothers and newborns from county-level hospitals with relatively large delivery volumes, thus the findings might not be generalizable to health facilities of other levels and provinces. Secondly, as data on SSC and breastfeeding practices were reported by postpartum mothers, recall bias may affect the precision of coverage indicators. Thirdly, the efficacy of EENC interventions may be underestimated if obstetrical health staff in control groups attended EENC-relevant technical training during the EENC implementation period. Finally, due to the unavailability of individual cases on health indicators, we could not link reported EENC practices with neonatal health impacts. The casual association between EENC practices and neonatal health impacts is worthy of further exploration.

## Conclusion

EEEC practices are feasible and have been successfully introduced to intervention hospitals of western China. EENC core interventions (except for kangaroo mother care) are routinely implemented in pilot health facilities, and the implementation is associated with reduced incidence risk of neonatal diarrhea and eye infection. The perceived usefulness of EENC interventions was identified by multiple stakeholders, who noted the willingness of staff to implement these interventions, the promotion of these interventions within hospital policy, the positive emotions experienced by postpartum mothers, and improvements in work-related support for health workers. The potential obstacles to EENC implementation mean concerted efforts should be made in China to meet the WHO target “at least 80% of facilities providing childbirth services implementing EENC”.

## Supplementary Information

**Additional file 1.**

## Data Availability

The datasets supporting the conclusions of this article are available from the corresponding author on reasonable request.
