# Supplementary material for: Effectiveness of early essential newborn care implementation in four counties of western China
Source: BMC Health Serv Res. 2022 Sep 21;22:1185. doi: 10.1186/s12913-022-08570-6 (PMC9494779; doi:10.1186/s12913-022-08570-6)
Supplement: Supplementary file 1 — Additional file 1. [file 12913_2022_8570_MOESM1_ESM.docx]

**Supplementary material for “Effectiveness of Early Essential Newborn Care implementation in four counties of Western China”**

This appendix provides more details of differences in coverage of Early Essential Newborn Care practices between intervention and control groups. The legends of online Figures and Tables are listed as bellows.

**CONTENT**

**Figure 1.** Difference in coverage of SSC (with 95% *CI*)

**Figure 2.** Difference in coverage of immediate SSC within 1min after birth (with 95% *CI*)

**Figure 3.** Difference in coverage of prolonged SSC for ≥90min (with 95% *CI*)

**Figure 4.** Difference in coverage of successful first-time breastfeeding with provision of SSC (with 95% *CI*)

**Figure 5.** Difference in any breastfeeding rate (with 95% *CI*)

**Figure 6.** Difference in coverage of early breastfeeding initiation within 60 min after birth (with 95% *CI*)

**Figure 7.** Difference in coverage of prolonged duration of first-breastfeeding for ≥ 30min (with 95% *CI*)

**Figure 8.** Difference in exclusive breastfeeding rate (with 95% *CI*)

**Figure 9.** Difference in coverage of no applied medicine to the umbilical cord (with 95% *CI*)

**Figure 10.** Difference in coverage of routine eye care (with 95% *CI*)

**Figure 11.** Difference in coverage of vitamin K_1_ administration (with 95% *CI*)

**Figure 12.** Thematic framework on effects of EENC implementation

**Table 1.** Group allocation and on-site survey time

**Table 2.** Difference in general coverage of EENC core interventions in four provinces according to endline survey

**Table 3.** Sample size of interviewees

(1.72, 10.81)

Sichuan

Ningxia

(46.68, 54.18)

(49.24, 65.45)

(92.13, 97.68)

(-0.03, 6.52)

Guizhou

Total

Qinghai

**Figure 1.** Difference in coverage of SSC (with 95% *CI*)

(83.94, 94.09)

(73.91, 87.70)

(-42.76, -25.49)

Qinghai

Total

Sichuan

Guizhou

Ningxia

(88.42, 96.82)

(57.97, 68.43)

**Figure 2.** Difference in coverage of immediate SSC within 1min after birth (with 95% *CI*)

Ningxia

Guizhou

Total

Qinghai

Sichuan

(69.65, 84.74)

(77.23, 89.33)

(52.13, 67.37)

(74.02, 80.86)

(85.92, 95.29)

**Figure 3.** Difference in coverage of prolonged SSC for ≥90min (with 95% *CI*)

Total

(3.97, 14.10)

(-5.74, -0.10)

(6.17, 19.61)

(29.04, 84.95)

(-18.73, 2.66)

Qinghai

Sichuan

Guizhou

Ningxia

**Figure 4.** Difference in coverage of successful first-time breastfeeding with provision of SSC (with 95% *CI*)

Guizhou

Total

Qinghai

Sichuan

Ningxia

(-2.45, 3.73)

(-3.54, 8.62)

(7.40, 15.62)

(-3.17, 7.36)

(2.46, 7.46)

**Figure 5.** Difference in any breastfeeding rate (with 95% *CI*)

(-21.42, -3.43)

Ningxia

(6.63, 14.37)

(16.94, 31.58)

(-2.24, 11.50)

(16.45, 28.26)

Total

Qinghai

Sichuan

Guizhou

**Figure 6.** Difference in coverage of early breastfeeding initiation within 60 min after birth (with 95% *CI*)

Sichuan

(-1.87, -18.90)

Total

Qinghai

(8.47, 19.16)

(18.74, 40.70)

(20.68, 39.82)

Guizhou

Ningxia

(-31.75, -16.71)

**Figure 7.** Difference in coverage of prolonged duration of first-breastfeeding for ≥ 30min (with 95% *CI*)

Total

Qinghai

Sichuan

Guizhou

Ningxia

(-0.64, 16.92)

(6.35, 24.13)

(47.87, 62.06)

(24.48, 33.06)

(11.59, 25.45)

**Figure 8.** Difference in exclusive breastfeeding rate (with 95% *CI*)

Total

(-91.17, -86.37)

(-60.88, -43.83)

Qinghai

Sichuan

(-99.93, -94.36)

Guizhou

(-99.98, -95.28)

Ningxia

(-100, -98.15)

合计

**Figure 9.** Difference in coverage of no applied medicine to the umbilical cord (with 95% *CI*)

(48.97, 65.60)

Total

Qinghai

Sichuan

Guizhou

Ningxia

(81.52, 87.34)

(100, 100)

(100, 100)

(-22.39, -4.39)

**Figure 10.** Difference in coverage of routine eye care (with 95% *CI*)

**Figure 11.** Difference in coverage of vitamin K_1_ administration (with 95% *CI*)

Total

Qinghai

Ningxia

Sichuan

Guizhou

(-0.67, 2.07)

(-2.47, 2.88)

(-1.79, 1.19)

(6.81, 11.88)

(34.12, 51.40)

**Figure 12. Thematic framework on effects of EENC implementation**

**Effect**

**Recognition**

**Policy**

**Work support**

**Health outcome**

**Sustainability**

**Emotion**

**Expertise**

**Hypothermia**

**Umbilical infection**

**Neonatal death**

**Happiness**

**Standardization**

**Cost**

**Workload**

**Parent-Child Relationship**

**Table 1.** Group allocation and on-site survey time

| On-site survey time | Province | Intervention Group | |  | Control Group | |
| --- | --- | --- | --- | --- | --- | --- |
|  |  | County | Health Facility |  | County | Health Facility |
| December, 2020 | Ningxia Hui Autonomous Region | Longde | People’s Hospital |  | Pengyang | People’s Hospital |
|  |  |  | Maternal and Child Health Care Hospital |  |  | Maternal and Child Health Care Hospital |
| March, 2021 | Guizhou Province | Qinglong | People’s Hospital |  | Puan | People’s Hospital |
|  |  |  | Maternal and Child Health Care Hospital |  |  | Maternal and Child Health Care Hospital |
| April, 2021 | Sichuan Province | Tongjiang | People’s Hospital |  | Nanbu | People’s Hospital |
|  |  |  | Maternal and Child Health Care Hospital |  |  | Maternal and Child Health Care Hospital |
| April, 2021 | Qinghai Province | Tongren | People’s Hospital |  | Gonghe | People’s Hospital |
|  |  |  |  |  |  | Hospital of Chinese Medicine |

**Table 2.** Difference in general coverage of EENC core interventions in four provinces according to endline survey

|  | Total (*N*=1298) | | | |  | Guizhou Province (n=421) | | | | |  | Qinghai Province (n=280) | | | | |  | Sichuan Province (n=282) | | | | |  | Ningxia Hui Autonomous Region (n=315) | | | |
| --- | --- | --- | --- | --- | --- | --- | --- | --- | --- | --- | --- | --- | --- | --- | --- | --- | --- | --- | --- | --- | --- | --- | --- | --- | --- | --- | --- |
|  | Intervention  (n=599) | Control  (n=699) | *χ*^2^ | *P* |  | Intervention  (n=150) | Control  (n=271) | *t*/*χ*^2^ | | *P* |  | Intervention  (n=137) | Control  (n=143) | *t*/*χ*^2^ | *P* | |  | Intervention  (n=152) | Control  (n=130) | *t*/*χ*^2^ | | *P* |  | Intervention  (n=160) | Control  (n=155) | *t*/*χ*^2^ | *P* |
| Any one practice | 599  (100.00) | 676  (96.71) | 24.805^＃^ | <0.001 |  | 150  (100.00) | 270  (99.63) | — | 1^*^ | |  | 137  (100.00) | 143  (100.00) | — | | — |  | 152  (100.00) | 130  (100.00) | — | — | |  | 160  (100.00) | 133  (85.81) | 24.415 | <0.001 |
| Any two practices | 590  (98.50) | 71  (10.16) | 1037.032^＃^ | <0.001 |  | 150  (100.00) | 1  (0.37) | 416.669 | <0.001 | |  | 137  (100.00) | 65  (45.45) | 103.582 | | <0.001 |  | 152  (100.00) | 3  (2.31) | 270.160 | <0.001 | |  | 151  (94.38) | 2  (1.29) | 273.094 | <0.001 |
| Any three practices | 587  (98.00) | 64  (9.16) | 1047.844^＃^ | <0.001 |  | 150  (100.00) | 0  (0.00) | 421.00 | <0.001 | |  | 137  (100.00) | 62  (43.36) | 109.188 | | <0.001 |  | 151  (99.34) | 2  (1.54) | 270.063 | <0.001 | |  | 149  (93.13) | 0  (0.00) | 273.905 | <0.001 |
| Any four practices | 517  (86.31) | 41  (5.86) | 875.213^＃^ | <0.001 |  | 142 (94.67) | 0  (0.00) | 387.119 | <0.001 | |  | 131  (95.62) | 41  (28.67) | 132.359 | | <0.001 |  | 133  (87.50) | 0  (0.00) | 215.285 | <0.001 | |  | 111  (69.38) | 0  (0.00) | 166.041 | <0.001 |
| All five  practices | 404  (67.45) | 0  (0.00) | 709.221^＃^ | <0.001 |  | 128 (85.33) | 0  (0.00) | 332.279 | <0.001 | |  | 100  (72.99) | 0  (0.00) | 162.368 | | <0.001 |  | 113  (74.34) | 0  (0.00) | 161.265 | <0.001 | |  | 63  (39.38) | 0  (0.00) | 76.289 | <0.001 |

*: Fisher exact probability

＃: Cochran-Mantel-Haenszel (CMH) with control variable as province

**Table 3.** Sample size of interviewees

|  | Total | |  | Guizhou | |  | Qinghai | |  | Sichuan | |  | Ningxia | |
| --- | --- | --- | --- | --- | --- | --- | --- | --- | --- | --- | --- | --- | --- | --- |
|  | Intervention | Control |  | Intervention | Control |  | Intervention | Control |  | Intervention | Control |  | Intervention | Control |
| Policymakers | 25 | 27 |  | 7 | 6 |  | 5 | 8 |  | 9 | 7 |  | 4 | 6 |
| Health staff | 45 | 49 |  | 9 | 6 |  | 6 | 12 |  | 18 | 16 |  | 12 | 15 |
| Postpartum mothers | 15 | 22 |  | 7 | 3 |  | 3 | 6 |  | 2 | 8 |  | 3 | 5 |
